# Novel strategy for primary epithelial cell isolation: Combination of hyaluronidase and collagenase I

**DOI:** 10.1111/cpr.13320

**Published:** 2022-08-03

**Authors:** Zhewen Hu, Yiming Chen, Min Gao, Xiaopei Chi, Ying He, Chenguang Zhang, Yue Yang, Yuman Li, Yan Lv, Ying Huang, Xuliang Deng

**Affiliations:** ^1^ Department of Geriatric Dentistry Peking University School and Hospital of Stomatology Beijing People's Republic of China; ^2^ Department of Oral Implantology, Guanghua School of Stomatology, Hospital of Stomatology, Guangdong Provincial Key Laboratory of Stomatology Sun Yat‐Sen University Guangzhou People's Republic of China; ^3^ Department of Prosthodontics, The First Clinical Division Peking University School and Hospital of Stomatology Beijing People's Republic of China; ^4^ Beijing Institute of Dental Research, Beijing Stomatological Hospital and School of Stomatology Capital Medical University Beijing People's Republic of China

## Abstract

**Objective:**

Different strategies for epithelial cell isolation significantly affect the viability and physiological properties of primary cells. Trypsin digestion, a conventional method, causes collateral damage owing to its strong digestive potential. To better preserve the physiological properties of epithelial tissues, we aimed to develop a modified method (hyaluronidase and collagenase I combination) for primary cell isolation.

**Method:**

We used conventional and modified methods to compare cell viability, morphology and stemness. Additionally, we investigated the passaging stability of epithelial cells and their capacity for organoid formation. Finally, we compared the two methods for isolating urothelial, oesophageal, lingual, and epidermal epithelial cells.

**Result:**

Gingival epithelial cells obtained using the modified method had higher viability, better morphology and stronger stemness than those obtained using the conventional method. Additionally, primary cells obtained using the modified method were stably passaged. Regarding organoid culture, adopting the modified method led to a significant increase in the growth rate and expression of the stem cell markers cytokeratin (CK)‐19 and Ki‐67. Furthermore, the modified method outperformed the conventional method for isolating urothelial, epidermal, oesophageal and lingual epithelial cells.

**Conclusion:**

We demonstrated that the combination of hyaluronidase and collagenase I outperformed trypsin in preserving the physiological properties of primary cells and organoid formation. The modified method could be broadly applied to isolate different types of epithelial cells and facilitate studies on organoids and tissue engineering.

## Graphical abstract

In terms of primary cell isolation, we used conventional (trypsin digestion) and modified (hyaluronidase and collagenase I combination digestion) methods to compare primary cell viability, morphology and stemness. Gingival epithelial cells obtained using the modified method had higher viability, better morphology and stronger stemness than those obtained using the conventional method. Regarding organoid culture, adopting the modified method led to a significant increase in the growth rate and expression of the stem cell markers.

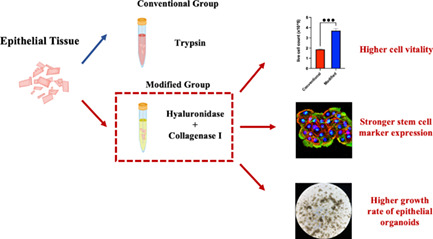

## 1 INTRODUCTION

The epithelium, which covers the surface of various tissues and organs, acts as a barrier against external stimuli and plays a pivotal role in tissue regeneration.^1^, ^2^, ^3^, ^4^ In vitro studies on epithelial tissues have provided a significant reference for clinical practices in aspects of tissue engineering, drug screening and host‐pathogen interactions.^5^, ^6^, ^7^, ^8^ Primary epithelial cell‐derived organoids are widely used in dermatology, urology, gastroenterology and several other research fields.^9^, ^10^, ^11^ The introduction of organoids has broadened the potential application prospects of primary epithelial cells and put forward higher demands for the preservation of their physiological properties.^12^, ^13^

Despite the broadening of their application scope, little improvement has been achieved in strategies for epithelial cell isolation.^14^ Explant culture and enzymatic digestion are the two most commonly applied methods for epithelial cell isolation.^15^, ^16^ The former approach is easier to operate and more cost‐effective; however, it is more time consuming and could lead to fibroblast contamination.^17^, ^18^, ^19^ In comparison, the enzymatic digestion approach possesses higher efficiency and specificity and is thereby more widely adopted.^16^, ^20^, ^21^, ^22^ The most commonly applied enzyme in previous studies was trypsin.^21^, ^23^ Degrading the intercellular junctions of epithelial tissues are more difficult than those of other tissues; therefore, a prolonged digestion period is required to obtain an adequate amount of cells.^3^, ^20^, ^21^ As a consequence, the isolation process may severely damage the primary cells. Therefore, despite the feasibility of trypsin digestion in establishing two‐dimensional cell culture systems, it cannot preserve physiological properties of the original tissue.^21^, ^23^ Previous studies have demonstrated that prolonged trypsin digestion affects cell viability and impairs surface proteins, some of which are critical stem cell markers.^23^, ^24^ When primary cell stemness is weakened, their proliferative activity and organoid‐forming ability severely decline.^25^ Therefore, an effective yet gentle approach must be developed to reduce the alteration of physiological properties, particularly stemness, of primary cells.^23^, ^26^

Collagenase, a *clostridium histolyticum‐*derived protease, is widely applied in primary cell isolation.^16^ Based on its substrate, collagenase is divided into six subtypes, among which collagenase I is used for epithelial cell isolation.^13^ Collagenase I, a less potent enzyme, causes less damage to primary cells during the digestion process; however, it requires an extended digestion time to obtain an adequate number of cells, which reduces efficiency.^27^ Hyaluronidase, an endogenous glycosidase, is synthesized in animals and microbes and is essential in cancer metastasis as it promotes cancer cell diffusion by increasing tissue permeability.^28^, ^29^ Moreover, in vivo studies on oral microbes have suggested that hyaluronidase can dissociate intercellular junctions in the sulcular eptihelium.^30^ Nevertheless, the capacity of collagenase and hyaluronidase combination in isolating epithelial cells remains to be investigated.

In this study, we introduced a novel strategy for primary epithelial cell isolation by replacing trypsin with a combination of hyaluronidase and collagenase I. In view of ethical issues and the accessibility of samples, we chose human gingiva as a representative of epithelial tissues.

## 2 MATERIALS AND METHODS

**FIGURE 1:**
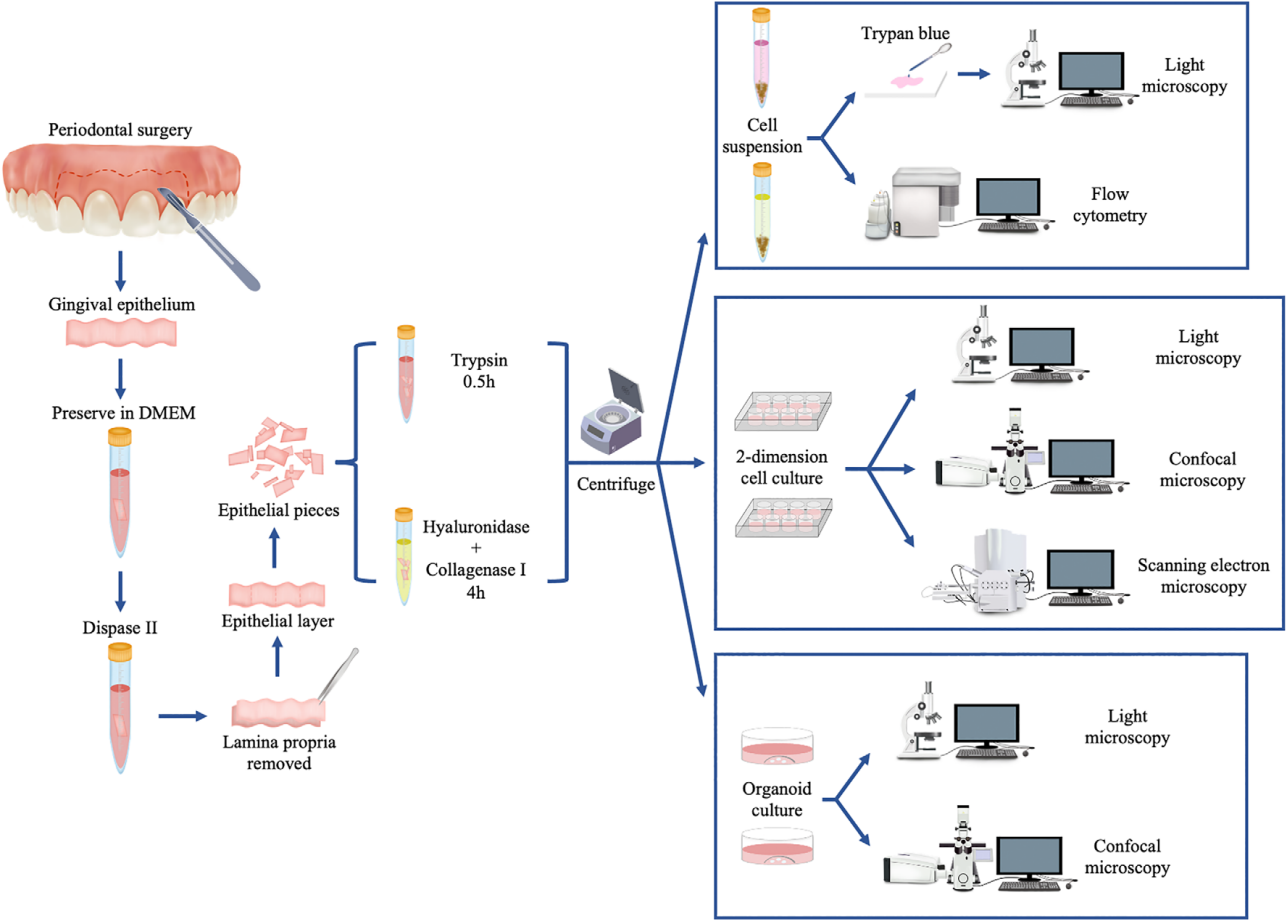
Experiment scheme depicting primary cell isolation and phenotypic validation.

We used trypsin (Gibco: 25200072) and a combination of hyaluronidase (Merck: H3506) and collagenase I (Gibco: 17018029) for epithelial cell isolation (Figure 1). According to the literature, the digestion time of trypsin should be limited to 30–40 min, whereas that of hyaluronidase and collagenase I ranges from 1 h to overnight.^13^, ^16^, ^21^, ^23^ We set the digestion time of hyaluronidase and collagenase I to 4 h, as suggested by most studies.^13^, ^16^ To control the variables across different groups, we set two different digestion times for each enzyme: 0.5 and 4 h. Consequently, we established four groups (Table 1). To control the variables, we ensured that all other conditions except for the enzymes were identical in the four groups.

**TABLE 1:** Methods applied for isolation of epithelial cells

Group	Method	Digestion time
1	0.125% Trypsin	37°C, 0.5 h
2		37°C, 4 h
3	1 mg/ml hyaluronidase + 2 mg/ml collagenase I	37°C, 0.5 h
4		37°C, 4 h

### 2.1 Epithelial tissue collection

Human gingival samples were collected during periodontal surgeries. A total of three participants were involved in the study. The epidermis, tongue, urothelium and oesophagus were obtained from SD rats (6 weeks, male, 160 g) after euthanasia (15 mg/kg pentobarbital) by surgical excision. The samples were washed with sterilized saline solution and preserved at 4°C in Dulbecco's Modified Eagle Medium (DMEM, Gibco: 12634010) supplemented with 5% penicillin/streptomycin solution (Gibco: 15140122).

### 2.2 Ethics statement

Gingival tissue collection was approved by the Human Ethics Committee of Peking University School and Hospital of Stomatology (Approval number: PKUSSIRB‐202168143). The donors who participated in our study volunteered and signed informed consent forms. The animal surgical procedure was approved by the Institutional Animal Care and Use Committee of the Peking University (Approval number: LA2022394). The SD rats were purchased from Beijing HFK Bioscience Co. Ltd. All efforts were made to minimize the suffering of animals.

### 2.3 Epithelial cell isolation

Epithelial tissues were transferred to a centrifuge tube containing 4 ml of Dispase II (2.5 mg/ml, Roche: 04942078001), which detached the epithelial layer from the lamina propria.^17^, ^21^, ^23^ Centrifuge tubes were maintained at 4°C for 6 h. Afterwards, the epithelial layer was peeled off using forceps and washed with a sterilized phosphate buffered saline (PBS). The epithelial tissue was equally segmented into four pieces and placed into the corresponding centrifuge tubes marked as Method 1, 2, 3, or 4 (Table 1). To increase digestion efficiency, the tissues were cut into 1 mm^2^ fragments. Thereafter, 2 ml of the enzyme was added to each centrifuge tube. The tubes were placed in an incubator at 37°C. The tissue suspension was sheared every 10 min using a 1000 μl pipette. Digestion was terminated by adding 4 ml of DMEM containing 5% foetal bovine serum (FBS; Gibco: 16140071). The suspension was blended with a pipette, strained over a 100 μm filter, and centrifuged at 400 × *g* for 5 min. In case of gingival epithelial cells, the supernatant was removed, and the sediment was resuspended in oral keratinocyte medium (OKM, Science Cell: 2611) for cell culture or in organoid‐qualified basement membrane extract (BME) matrix (R&D Systems: 3533‐001‐02) for organoid culture.

### 2.4 Cell inoculation and passaging

According to the requirements of follow‐up experiments, primary epithelial cells were cultured in different culture dishes (Table 2). To control the variables, the number of cells inoculated in each well was identical in the four groups. Culture dishes were kept in incubators (37°C, 5% CO_2_).

**TABLE 2:** Culture dish and cell inoculation

Experiment	Culture dish	Number of cells per well
Light microscopy	24 well plate	2 × 10^5^
Immunofluorescence	24 well confocal plate	2 × 10^5^
Scanning electron microscopy	24 well plate with glass coverslip	2 × 10^5^

Cell passaging was performed when the cells reached 80% confluency. Adherent epithelial cells were detached by adding trypsin. Digestion was carefully monitored and terminated using DMEM supplemented with 10% FBS when 80% of cells were detached from the plate. Cell suspension was centrifuged at 1000 rpm for 5 min. The supernatant was removed, and the sediment was resuspended in OKM. The cells were then cultured on new plates. The intervals between passages were documented.

### 2.5 Organoid culture

Primary epithelial cells resuspended in the BME matrix were plated in preheated 24‐well plates (50 μl/well). Afterwards, the plates were inverted and placed in an incubator at 37°C for 30 min to induce BME matrix solidification. Subsequently, pre‐warmed organoid medium (Table 3) was added to each well. The culture medium was changed every 2 days, and 10 μmol/L Rho‐associated kinase inhibitor Y‐27632 (MCE: HY‐10071) was added for the first week as a supplement to facilitate organoid outgrowth.

**TABLE 3:** Composition of organoid culture medium

Reagent	Product model	Concentration
DMEM/F12	Gibco 12634‐010	
Penicillin‐Streptomycin	Gibco 15140‐122	100 U/ml
Hepes	Gibco 15630‐080	10 mmol/L
GlutaMax	Gibco 35050‐061	1×
B‐27 supplement	Gibco 17504‐044	1×
Human R‐Spondin 1	R&D 4645‐RS‐100/CF	10 ng/ml
Human Noggin	PeproTech 250‐38‐5	10 ng/ml
Human EGF	PeproTech AF‐100‐15	50 ng/ml
Nicotinamide	Sigma N0636‐100	10 mmol/L
A83‐01	MCE HY‐10432	1 μmol/L
Forskolin	R&D 1099	1 μmol/L
CHIR99021	Sigma SML1046	0.5 μmol/L
N‐acetyl‐L‐cysteine	Sigma A9165	1 mmol/L

### 2.6 Comparison of different digestion methods

#### 2.6.1 Cell count

Cell suspension (20 μl) from each group was collected and mixed with equal volumes of trypan blue. The total number of cells and number of live cells were counted using an automatic cytometer. Cell viability = live cell number/total cell number.

#### 2.6.2 Flow cytometry

Following isolation, the cells were resuspended in 3% bovine serum albumin (BSA), and blank medium was used as control. Primary antibodies (diluted at 1:20 in 3% BSA) cytokeratin (CK)‐19 (Abcam: 7754) and Ki‐67 (Abcam: 16667) were added to all tubes, except for the blank control, and incubated at 4°C for 30 min. The tubes were shaken gently every 10 min. Afterwards, the tubes were centrifuged at 1000 rpm for 5 min, washed twice with 3% BSA, and resuspended in 3% BSA. Secondary antibodies (diluted at 1:250 in PBS) were added to all tubes and incubated at 4°C for 30 min in the dark. The tubes were gently shaken every 10 min, centrifuged at 1000 rpm for 5 min, and washed twice with 3% BSA. Prior to testing, the cells were resuspended in 300 μl of 3% BSA.

#### 2.6.3 Scanning electron microscopy

Epithelial cells cultured on glass coverslips were fixed with glutaraldehyde (Sigma‐Aldrich, 1.04239) for 20 min. Fixed cells were washed twice with PBS, and dehydrated using gradient ethanol (30%, 50%, 70%, 80%, 90%, 95% and 100%). Prior to observation, glass coverslips were placed in a vacuum drying oven overnight to eliminate moisture.

#### 2.6.4 Immunofluorescence analysis

Epithelial cells or organoids cultured on confocal plates (Cellvis, P24‐1.5H‐N) were washed with PBS and fixed with 4% paraformaldehyde for 20 min. Fixed cells were washed with PBS, permeabilized with 0.5% Triton (diluted in 3% BSA) for 5 min, and washed again with PBS. Afterwards, 3% BSA was added for 30 min to block non‐specific binding sites. Samples were incubated with primary antibodies (diluted at 1:200 in 1% BSA) at 4°C for 8 h. Thereafter, the cells were washed with PBS and incubated with secondary antibodies (diluted at 1:250 in PBS) for 1 h at room temperature. Cell nuclei were stained with DAPI (diluted at 1:100 in PBS), and the cytoskeleton was stained with fluorescein isothiocyanate‐phalloidin (diluted at 1:200 in PBS). The plates were incubated at 4°C and covered with ice‐cold PBS prior to examination.

### 2.7 Statistics

Experiments for each group were performed in triplicate. All figures are representative of data sets. Statistical analysis was performed using SPSS (version 26), and diagrams were plotted using GraphPad Prism (version 9.0). Analysis between more than two sample groups was performed by a one‐way unstacked ANOVA and post‐hoc LSD testing. Analysis between two paired samples was performed by a two‐tailed unpaired Student's *t*‐test. ns: not statistically significant; *p < 0.05, **p < 0.01 and ***p < 0.001. *p* < 0.05 was considered statistically significant.

## 3 RESULTS

### 3.1 Primary gingival epithelial cells obtained using modified method had higher viability and better morphology

**FIGURE 2:**
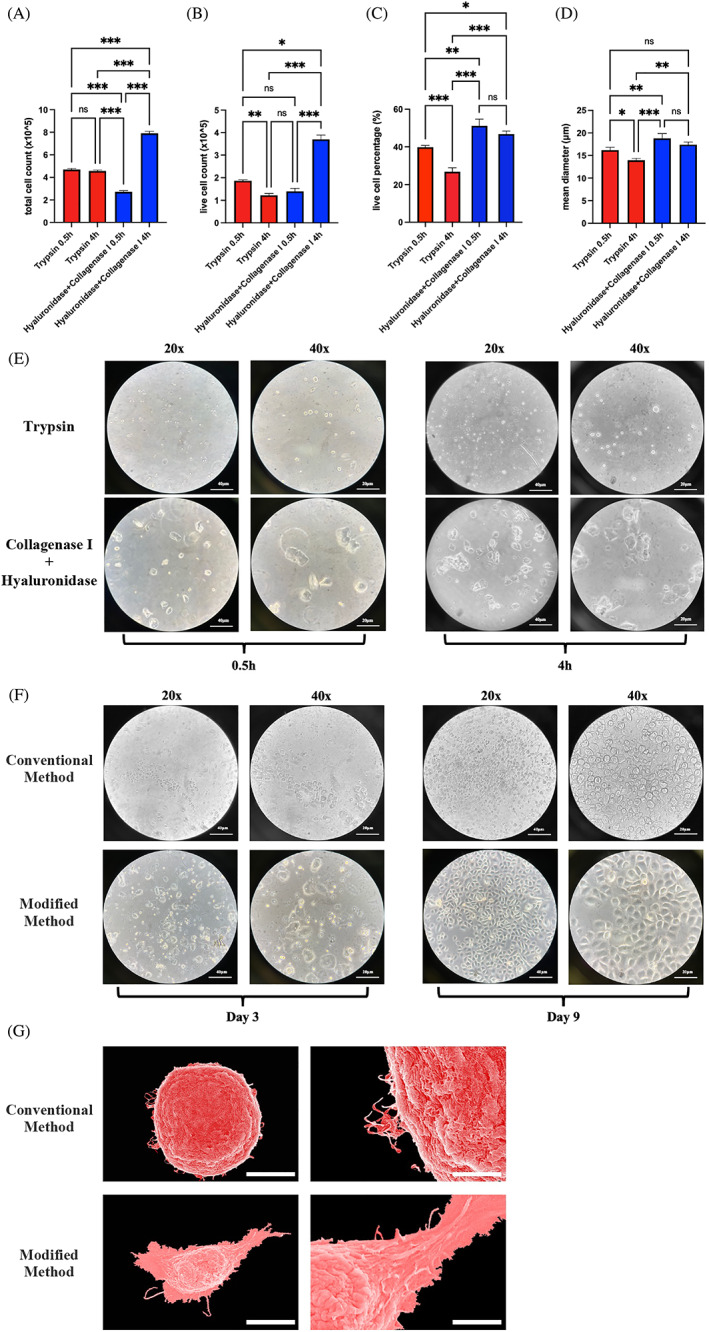
Primary epithelial cells obtained using modified isolation had higher viability and better morphology. (A) The total number of cells obtained using four methods (Method 2 yielded the smallest number of cells, whereas Method 4 yielded the greatest number of cells, *p* < 0.05; and no significant difference was observed in the number of total cells obtained using Methods 1 and 2, *p* > 0.05, *n* = 3). (B) The number of live cells obtained using four methods (Method 4 yielded a significantly greater number of cells compared with all other methods, *p* < 0.05, *n* = 3; whereas Method 2 yielded the least number of cells). (C) Live cell percentages obtained using four methods (Methods 3 and 4 yielded significantly higher live cell percentages than Methods 1 and 2 did, *p* < 0.05, and no significant difference was observed between Methods 3 and 4, *p* > 0.05, *n* = 3). (D) The mean cell diameter obtained using four methods (Method 3 yielded the largest cell diameter, followed by Methods 4 and 1, and Method 2 yielded a significantly smaller cell diameter than the other methods, *n* = 3). (E) Light microscopy images of cells obtained using four methods on the first day of culture show that cells isolated using collagenase I and hyaluronidase had a larger diameter and better morphology (scale bars, 40 and 20 μm). (F) Light microscopy images of cells obtained using the conventional and modified methods on Days 3 and 9 of culture show a better morphology and higher confluency in the modified group (scale bars, 40 and 20 μm). (G) Scanning electron microscopy of cells obtained using the conventional and modified methods on Day 6 of culture shows that cells in the modified group had a larger diameter and more distinct spreading of pseudopods (scale bars, 5 and 1.5 μm for conventional group; 10 and 3 μm for modified group). *p < 0.05, **p < 0.01, and ***p < 0.001. *p* < 0.05 was considered statistically significant.

We used the trypan blue assay to evaluate the efficiency of different methods. Extending the digestion time significantly increased the number of cells, particularly live cells, obtained using collagenase I and hyaluronidase digestion (Figure 2A,B). Importantly, this digestion did not significantly reduce cell viability (Figure 2C). Additionally, the mean diameter of primary cells isolated using collagenase I and hyaluronidase was larger than that of primary cells isolated using trypsin (Figure 2D). Thus, trypsin digestion shrank the primary epithelial cells more.

Light microscopy on the first day of culture revealed that primary cells isolated using collagenase I and hyaluronidase had a better morphology than that of primary cells isolated using trypsin. Notably, the former resembled slab stones, whereas the latter were mostly round or oval shaped (Figure 2E).

The optimal digestion time of trypsin should be limited to 30 min, as prolonged digestion leads to a sharp decline in cell viability, whereas that of hyaluronidase and collagenase I can be extended to 4 h. Therefore, we chose Methods 1 and 4 for further analysis. The former is referred to as the conventional method, whereas the latter is referred to as the modified method.

Regarding cell morphology, light microscopy revealed that primary cells obtained using the modified method had higher confluency and better morphology than primary cells obtained using the conventional method (Figure 2F). Scanning electron microscopy revealed that the mean cell diameter in the conventional group was approximately 15 μm, whereas that in the modified group was approximately 20 μm. This divergence was due to cell spreading, indicated by filopodia stretching which was more evident in the modified group (Figure 2G). Because filopodia protrusions are correlated with cell migration, primary cells obtained using the modified method had a stronger migration capacity than cells obtained using the conventional method.^31^

### 3.2 Modified method better preserved primary epithelial cell stemness during isolation

**FIGURE 3:**
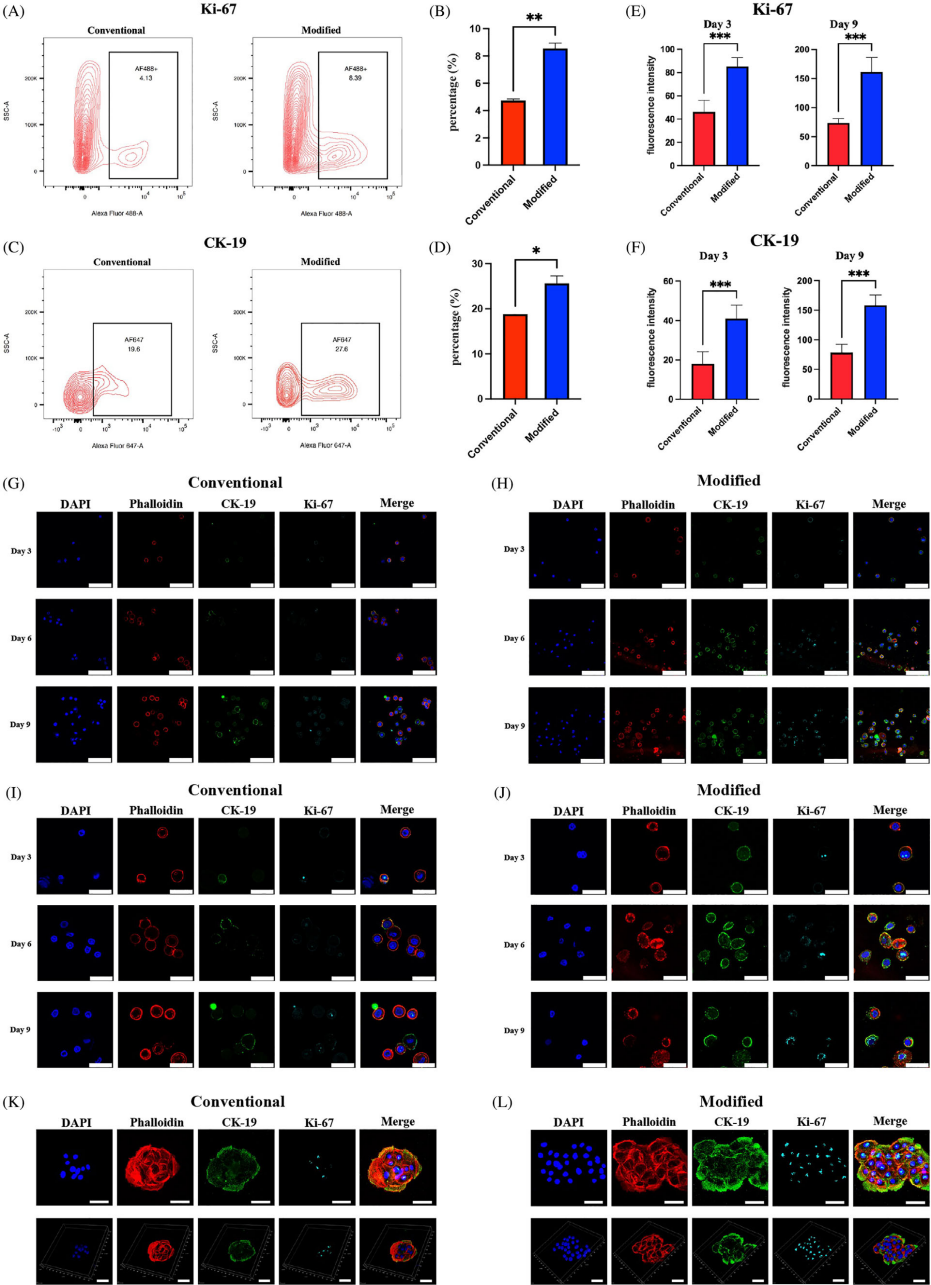
Primary epithelial cells obtained using modified isolation show more distinct stem cell features. (A and C) Counter chart depicting the percentage of (A) Ki‐67– and (C) CK‐19–positive cells in the conventional and modified groups identified using FCM. (B and D) Bar chart depicting the percentage of (B) Ki‐67– and (D) CK‐19–positive cells in the conventional and modified groups identified using FCM (the modified method yielded a significantly greater number of both Ki‐67– and CK‐19–positive cells compared with the conventional method, *p* < 0.05, *n* = 3). (E and F) Bar chart depicting the fluorescence intensity of CK‐19– and Ki‐67–positive cells on Days 3 (E) and 9 (F) of culture in the conventional and modified groups (the modified method yielded a significantly higher expression level of both Ki‐67– and CK‐19–positive cells on Day 3 and Day 9 compared with the conventional method, *p* < 0.05, *n* = 3). (G and H) Immunofluorescence (IF) images depicting CK‐19 and Ki‐67 expression on Days 3, 6 and 9 of cell culture in the conventional (G) and modified (H) groups (scale bar, 50 μm). (I and J) IF images depicting CK‐19 and Ki‐67 expression on Days 3, 6 and 9 of cell culture in the conventional (I) and modified (J) groups (scale bar, 25 μm). (K and L) IF images and three‐dimensional reconstruction of primary epithelial cells during colony formation obtained using the conventional (K) and modified (L) methods (scale bar, 25 μm). *p < 0.05, **p < 0.01, and ***p < 0.001. *p* < 0.05 was considered statistically significant.

We performed flow cytometry (FCM) immediately after cell isolation to examine divergence in the stem cell percentage. Primary cells were labelled with CK‐19, an epithelial stem cell/basal cell marker, and Ki‐67, a broadly applied stem cell marker. The former is predominantly found in the cell membrane, while the latter is located mainly in the nucleus.^14^, ^32^, ^33^, ^34^, ^35^ Primary cells in the modified group exhibited a higher percentage of CK‐19– and Ki‐67–positive cells than those in the conventional group, *p* < 0.05 (Figure 3A–D). Because the same piece of tissue was used for both methods, we determined that the modified method inflicted less damage to stem cells.

Immunofluorescence (IF) assays revealed divergence in CK‐19 and Ki‐67 expression levels between the two groups at different time points during cell culture. IF quantitation indicated that among CK‐19– and Ki‐67–positive cells, those in the modified group showed greater fluorescence intensity than their counterparts in the conventional group, *p* < 0.05 (Figure 3E,F). Moreover, IF analysis demonstrated that primary cells in the modified group exhibited a higher percentage of CK‐19– and Ki‐67–positive stem cells, which was consistent with the FCM results (Figure 3G–J). Upon cell colony formation, stem cell markers remained highly expressed in the modified group (Figure 3K,L).

### 3.3 Primary epithelial cells obtained using modified method were stably passaged

**FIGURE 4:**
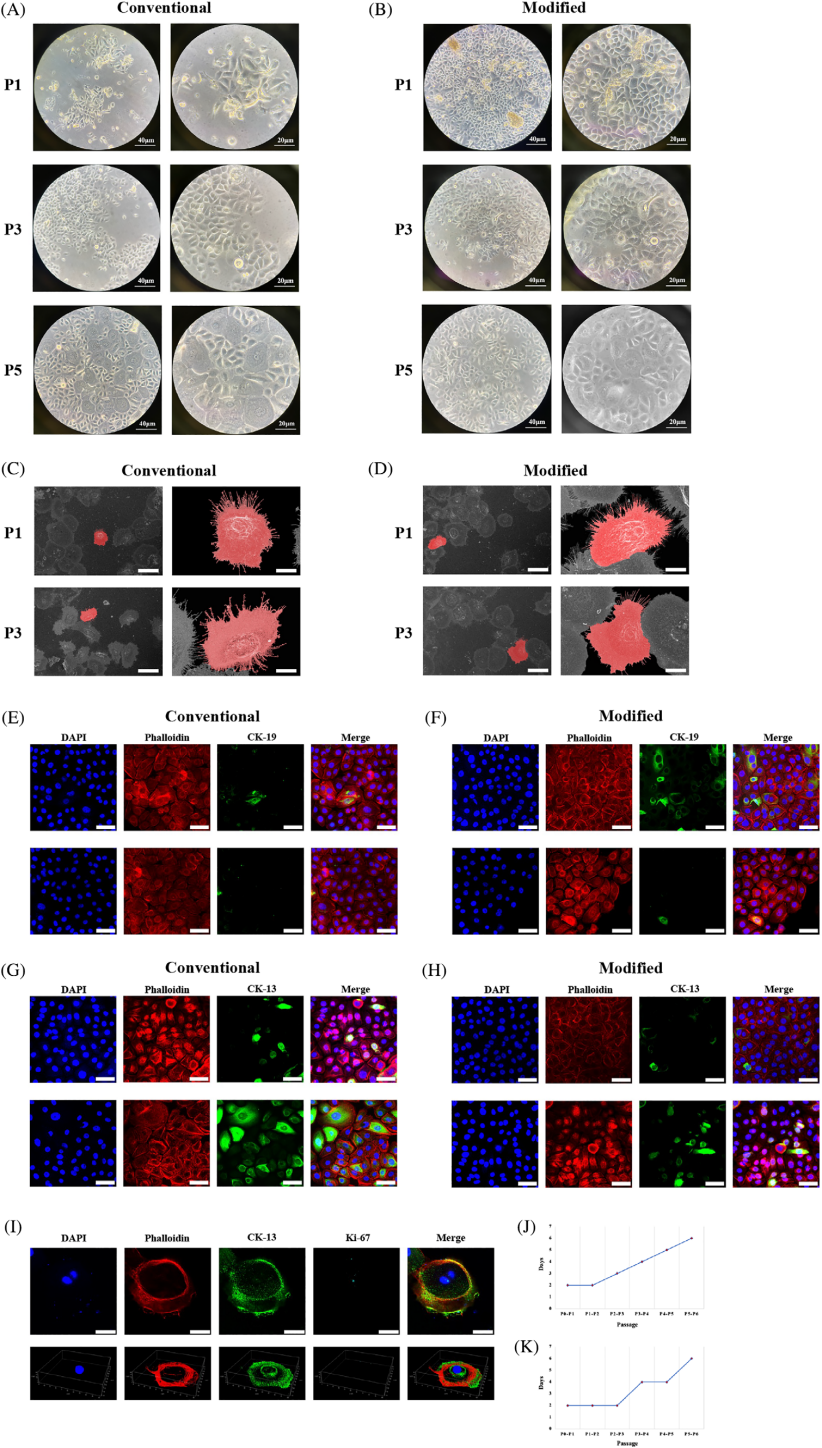
Primary epithelial cells obtained using modified isolation were stably passaged. (A and B) Light microscopy images of P1, P3 and P5 cells in the conventional (A) and modified (B) groups (scale bars, 40 and 20 μm). (C and D) Scanning electron microscopy images depicting the anchorage structures of P1 and P3 cells in the conventional (C) and modified (D) groups (scale bars, 5 and 2.5 μm). (E and F) Immunofluorescence (IF) images depicting CK‐19 expression in P1 and P3 cells in the conventional (E) and modified (F) groups (scale bar, 50 μm). (G and H) IF images depicting CK‐13 expression in P1 and P3 cells in the conventional (G) and modified (H) groups (scale bar, 50 μm). (I) IF images and three‐dimensional structure of P5 cells obtained in the modified group depicting the giant cell structure (scale bar, 50 μm). (J) Intervals of cell passaging between P0 and P6 in the control group. (K) Intervals of cell passaging between P0 and P6 in the modified group.

Primary epithelial cells from both groups were sub cultured to examine passaging stability. No significant difference in cell morphology was detected within the first three passages (Ps). Specifically, mean epithelial cell diameter remained consistent, and the cell contour resembled slab stones (Figure 4A,B). Additionally, cell stretching and attachment indicated by pseudopods remained essentially identical from P1 to P3 (Figure 4C,D). However, after P4, epithelial cells gradually lost morphology homogeneity. Despite the heterogeneity of the cell contour, giant cells characterized by a significant increase in diameter and dark particle accumulation in the nucleus constituted a fair percentage.

Despite consistent morphology, marker protein expression varied after cell passaging. We observed a marked reduction in the expression of the stem cell marker CK‐19 in P1–P3 cells (Figure 4E,F). Moreover, the expression of CK‐13, a differentiation‐related membrane marker, significantly increased from P1 to P3 (Figure 4G–I). Therefore, the percentage of stem cells decreased, whereas that of differentiated cells increased after passaging.

Noticeably, the growth rate of epithelial cells remained stable within P2–P3 but eventually decreased after further passaging, which was reflected by longer intervals between cell Ps (Figure 4J,K). After P5, we observed a prominent decrease in the growth rate, and epithelial cells gradually lost their passaging ability.

### 3.4 Gingival epithelial organoids obtained using modified method showed a higher growth rate and stem cell marker expression

**FIGURE 5:**
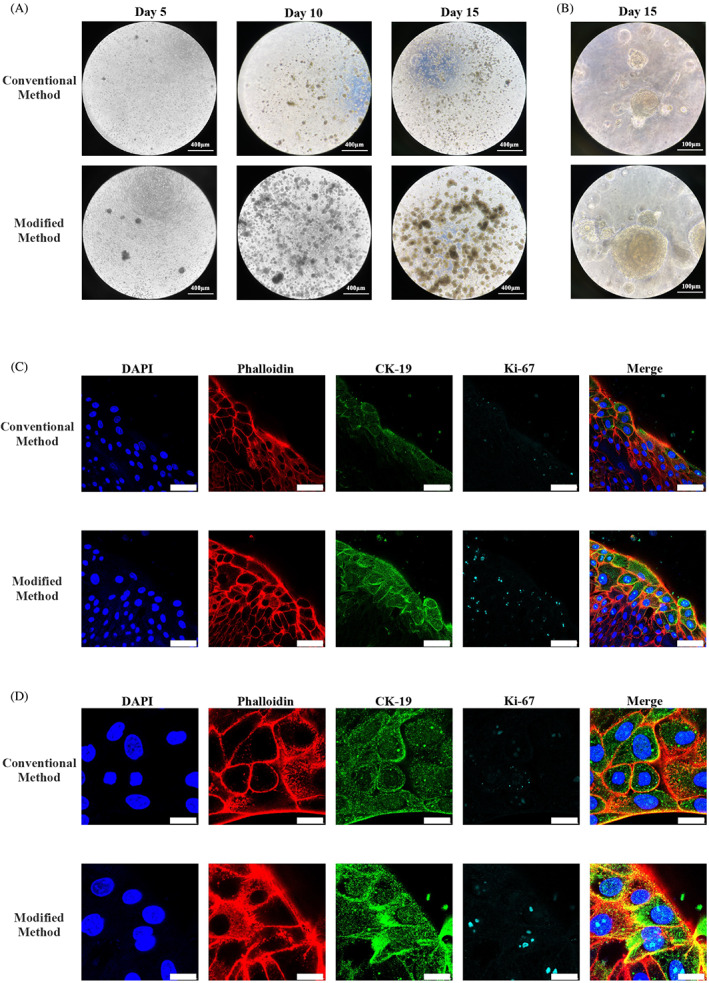
Primary epithelial cells obtained using modified isolation formed gingival epithelial organoids. (A and B) Light microscopy images of gingival epithelial organoids derived from primary cells obtained using the two methods on Days 5, 10 and 15 (scale bar, 400 and 100 μm). (C and D) Immunofluorescence image depicting the comparison of Ki‐67 and CK‐19 expression levels in gingival epithelial organoids derived from the control and modified groups (scale bar, 50 and 10 μm).

In both groups, round and oval organoids grew from epithelial cells and formed multilayer structures that resembled a stratified epithelium. Despite similar morphology, we observed significant variation in the growth rate between the two groups. Specifically, light microscopy revealed that organoids in the modified group were larger in size and greater in number than their counterparts in the conventional group (Figure 5A,B). Moreover, organoids in the modified group also showed higher expression of the stem cell markers CK‐19 and Ki‐67 (Figure 5C,D).

Thus, primary epithelial cells obtained using the conventional and modified methods formed organoids; however, the latter method yielded higher growth rates and stronger stem cell properties.

### 3.5 Modified method outperformed conventional method in epidermal, gastric and urothelial epithelial cell isolation

**FIGURE 6:**
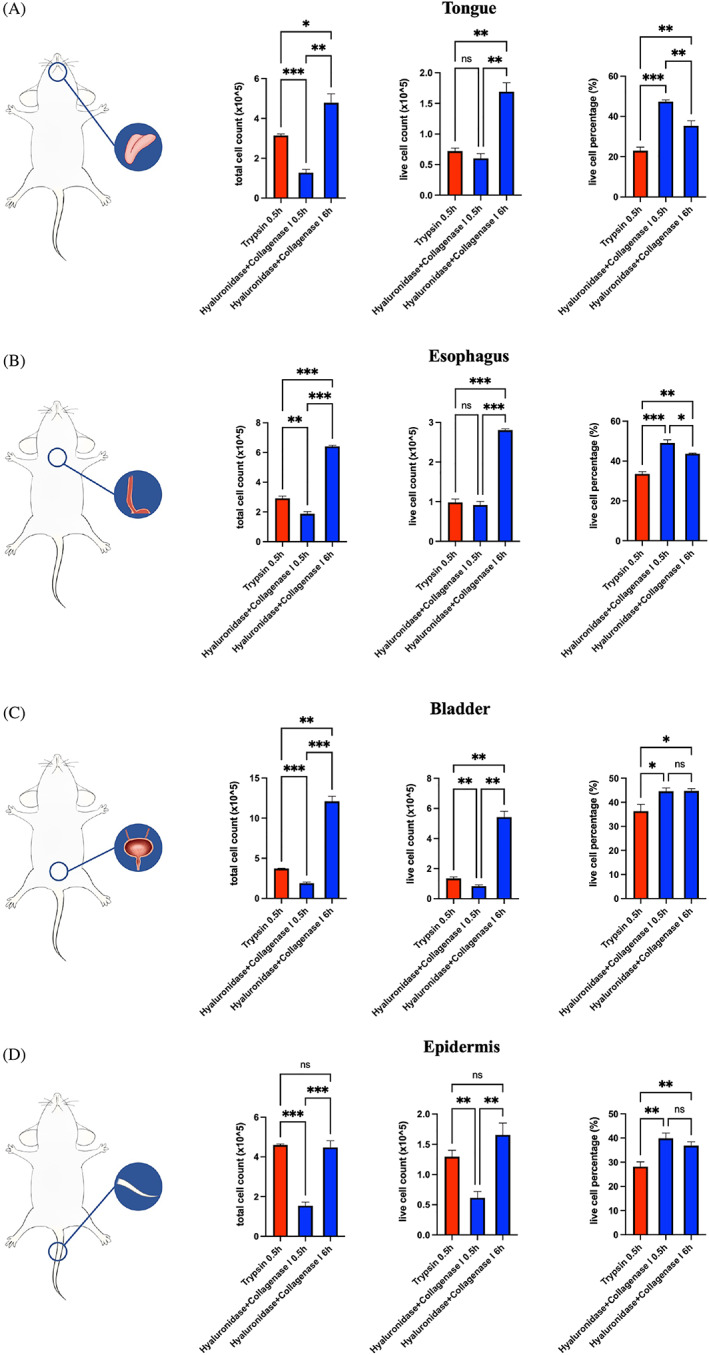
Modified method outperformed conventional method in rat epidermal, lingual, urothelial and oesophageal epithelial cell isolation in terms of cell viability and live cell count. (A–D) Bar chart depicting the total number of cells (left), number of live cells (middle) and live cell percentage (right) obtained using different methods of isolating murine epidermal (A), bladder (B), oesophageal (C) and lingual (D) cells (*n* = 3). *p < 0.05, **p < 0.01, and ***p < 0.001. *p* < 0.05 was considered statistically significant.

In addition to isolating gingival epithelial cells, our modified method was generalized to include epidermis, urothelium, tongue and oesophagus digestion. The modified method outperformed the conventional method in terms of the viability and number of live cells isolated from all four tissues, *p* < 0.05, *n* = 3 (Figure 6A–D). Notably, the modified method had more significant advantages in urothelial and oesophageal epithelium isolation than in lingual or epidermal epithelium isolation. Specifically, regarding urothelial and oesophageal epithelial cell isolation, the modified method yielded several times more live cells than the conventional method did (Figure 6B,C). This divergence was not as significant regarding epidermal and lingual cell isolation.

## 4 DISCUSSION

In vitro cultured epithelial cells are broadly applied in biomedical studies.^1^, ^2^, ^3^, ^4^ Consequently, primary epithelial cell acquisition has attracted increasing attention.^21^, ^23^ Trypsinization, the most commonly applied method for obtaining epithelial cells, has been broadly used in studies based on two‐dimensional cell cultures.^21^ However, despite its convenience, the simulation of in vivo conditions using two‐dimensional cultured cells is unsatisfactory, as they cannot maintain genomic stability during long‐term amplification.^13^, ^36^ The introduction of organoids has overcome several shortcomings of two‐dimensional cell lines and greatly facilitated studies on personalized therapy, drug toxicity and high‐throughput screening.^37^, ^38^ The broad application of organoids has set higher demands for primary epithelial cell isolation, as retaining stemness is essential to establish organoid models.^13^

We proposed a novel strategy for enzymatic isolation of primary epithelial cells by replacing trypsin with collagenase I and hyaluronidase. Generally, our modified method better preserved the physiological properties of original epithelial tissues. Particularly, collateral damage to primary cells was reduced by applying the modified method, which was reflected in cell viability and morphology. Moreover, FCM and IF assays confirmed that the modified method better preserved primary cell stemness. Consequently, organoids obtained using the modified method exhibited a faster growth rate. Furthermore, we demonstrated that primary cells obtained using the modified method were stably passaged, which is crucial for in vitro experiments and consistent with previous studies.^26^, ^39^ Ultimately, we generalized the modified method to include epidermal, lingual, gastric and urothelial epithelial cell isolation.

Studies on epithelial cell isolation have mostly focused on digestion efficiency, cell viability and cell morphology. We revealed that after P1, differences in cell morphology and proliferation rates narrowed between the two groups. Because studies on two‐dimensional cell lines typically adopt P2 or P3 epithelial cells, the conventional method is feasible, which might be why trypsinization has been predominantly employed in previous studies.^19^, ^26^ Nevertheless, primary epithelial cells are indispensable in organoid cultures and tissue engineering. Since organoid formation is highly dependent on stem cell state, collateral damage caused by digestion could affect organoid phenotype. Therefore, the conventional method has inevitable shortcomings when used for organoid construction, which might be why the success rate of organoid formation was relatively low in previous studies.^32^ Generally, stem cell state is critical for organoid formation, as phenotypic differences in organoids can be attributed to differences in the stemness of primary cells obtained using the two methods.^13^, ^32^ Additionally, cell passaging assay indicated that epithelial cell stemness diminished markedly after passaging. Thus, P3 cells might not be ideal for use in experiments regarding epithelial stem cells.

Owing to stemness preservation, our novel method significantly accelerated the growth rate of organoids. Since personalized therapy sets higher demands for the efficiency of organoid formation, our modified method might be of great significance in clinical settings, as it reduced the time of organoid formation, which reduced the timeline of in vitro experiments.^36^ Because some cancer cells are epithelial‐derived, our modified method might also be applied in digesting cancer tissues, which should be further researched.^40^, ^41^ The application scope of our method could be significantly expanded once it is applied to cancer cell isolation. Moreover, as cancer and stem cell markers share similar expression patterns under certain circumstances, investigating whether our modified strategy is also superior in preserving cancer tissue phenotype would be relevant.^42^, ^43^

Regarding the isolation of different epithelial cell types, we noticed that our novel strategy was better in digesting non‐keratinized epithelium, such as the oesophagus and urothelium. One possible reason is that the stratum corneum, a protective layer against external stimuli, decreased enzymatic digestion.^33^ Since the digestive capacity of the modified method was weaker than that of the conventional method, the stratum corneum had a more significant impact on the modified method. Consequently, the counteraction of the stratum corneum narrowed the gap between the two methods. Notably, epithelial structures vary between different epithelium types.^44^ Therefore, required isolation conditions may vary for different tissues, which remains to be elucidated. Further research is required to examine the differences in protein expression and function between the modified and conventional groups.

The popularization of organoids and tissue engineering has increased the demand for primary epithelial cell isolation, thereby emphasizing the importance of preserving the physiological state of original tissue. In addition to focusing on efficiency, further research on isolation methods should highly focus on preserving primary cell stemness and proliferation activity. Notably, compared to IF analysis and FCM, high‐throughput sequencing might provide a more comprehensive understanding of the divergence in gene expression patterns between primary cells and organoids obtained using the two methods.

## 5 CONCLUSION

In summary, we proposed a novel strategy for the enzymatic isolation of primary epithelial cells by replacing trypsin with hyaluronidase and collagenase I; the isolated cells exhibited higher cell viability, better cell morphology, and stronger stemness than those obtained by trypsinization did. Moreover, the modified method significantly shortened the growth cycle of and enhanced stem cell marker expression in organoid cultures. Thus, our novel strategy might facilitate studies on in vitro cultured epithelial cells and organoids.

## AUTHOR CONTRIBUTIONS

Zhewen Hu and Yiming Chen contributed equally to this work as co‐first authors. Ying Huang and Xuliang Deng conceived the idea for the study. Zhewen Hu, Yiming Chen, Min Gao, Xiaopei Chi and Ying He collected the data. Zhewen Hu, Yiming Chen, Chenguang Zhang, Yue Yang, Yuman Li and Yan Lv, discussed, and contributed to the final design of the study. Zhewen Hu and Yiming Chen wrote the first draft of the manuscript with significant assistance from Ying Huang and Xuliang Deng. All the authors contributed to the completion and revision of the manuscript.

## CONFLICT OF INTEREST

The authors declare that the research was conducted in the absence of any commercial or financial relationships that could be construed as a potential conflict of interest.

## Data Availability

The data used to support the findings of this study are available from the corresponding author upon request.

## References

[1] Takahashi N , Sulijaya B , Yamada‐Hara M , Tsuzuno T , Tabeta K , Yamazaki K . Gingival epithelial barrier: regulation by beneficial and harmful microbes. Tissue Barriers. 2019;7(3):e1651158. 31389292 10.1080/21688370.2019.1651158 PMC6748373

[2] Groeger SE , Meyle J . Epithelial barrier and oral bacterial infection. Periodontol 2000. 2015;69(1):46‐67. 26252401 10.1111/prd.12094

[3] Parrish AR . The impact of aging on epithelial barriers. Tissue Barriers. 2017;5(4):e1343172. 28686506 10.1080/21688370.2017.1343172 PMC5788442

[4] Takiishi T , Fenero CIM , Câmara NOS . Intestinal barrier and gut microbiota: shaping our immune responses throughout life. Tissue Barriers. 2017;5(4):e1373208. 28956703 10.1080/21688370.2017.1373208 PMC5788425

[5] Hyun DW , Kim YH , Koh AY , et al. Characterization of biomaterial‐free cell sheets cultured from human oral mucosal epithelial cells. J Tissue Eng Regen Med. 2017;11(3):743‐750. 25407749 10.1002/term.1971

[6] Kim J , Hegde M , Jayaraman A . Co‐culture of epithelial cells and bacteria for investigating host‐pathogen interactions. Lab Chip. 2010;10(1):43‐50. 20024049 10.1039/b911367c

[7] Castro‐Muñozledo F . Corneal epithelial cell cultures as a tool for research, drug screening and testing. Exp Eye Res. 2008;86(3):459‐469. 18191836 10.1016/j.exer.2007.11.017

[8] Butler CR , Hynds RE , Gowers KH , et al. Rapid expansion of human epithelial stem cells suitable for airway tissue engineering. Am J Respir Crit Care Med. 2016;194(2):156‐168. 26840431 10.1164/rccm.201507-1414OC PMC5003214

[9] Sugimoto S , Kobayashi E , Fujii M , et al. An organoid‐based organ‐repurposing approach to treat short bowel syndrome. Nature. 2021;592(7852):99‐104. 33627870 10.1038/s41586-021-03247-2

[10] Lee SH , Hu W , Matulay JT , et al. Tumor evolution and drug response in patient‐derived organoid models of bladder cancer. Cell. 2018;173(2):515‐528.e17. 29625057 10.1016/j.cell.2018.03.017 PMC5890941

[11] Lee J , Koehler KR . Skin organoids: a new human model for developmental and translational research. Exp Dermatol. 2021;30(4):613‐620. 33507537 10.1111/exd.14292 PMC8265774

[12] Lee GY , Kenny PA , Lee EH , Bissell MJ . Three‐dimensional culture models of normal and malignant breast epithelial cells. Nat Methods. 2007;4(4):359‐365. 17396127 10.1038/nmeth1015 PMC2933182

[13] Driehuis E , Kretzschmar K , Clevers H . Establishment of patient‐derived cancer organoids for drug‐screening applications. Nat Protoc. 2020;15(10):3380‐3409. 32929210 10.1038/s41596-020-0379-4

[14] Karakasheva TA , Kijima T , Shimonosono M , et al. Generation and characterization of patient‐derived head and neck, oral, and esophageal cancer organoids. Curr Protoc Stem Cell Biol. 2020;53(1):e109. 32294323 10.1002/cpsc.109 PMC7350550

[15] Kedjarune U , Pongprerachok S , Arpornmaeklong P , Ungkusonmongkhon K . Culturing primary human gingival epithelial cells: comparison of two isolation techniques. J Craniomaxillofac Surg. 2001;29(4):224‐231. 11562092 10.1054/jcms.2001.0229

[16] Kloskowski T , Uzarska M , Gurtowska N , et al. How to isolate urothelial cells? Comparison of four different methods and literature review. Hum Cell. 2014;27(2):85‐93. 24368576 10.1007/s13577-013-0070-y

[17] Rakhorst HA , Tra WM , Posthumus‐van Sluijs SJ , et al. Mucosal keratinocyte isolation: a short comparative study on thermolysin and dispase. Int J Oral Maxillofac Surg. 2006;35(10):935‐940. 16965903 10.1016/j.ijom.2006.06.011

[18] Oda D , Watson E . Human oral epithelial cell culture I. Improved conditions for reproducible culture in serum‐free medium. In Vitro Cell Dev Biol. 1990;26(6):589‐595. 2358421 10.1007/BF02624208

[19] Bryja A , Popis M , Borowiec B , et al. Overview of the different methods used in the primary culture of oral mucosa cells. J Biol Regul Homeost Agents. 2019;33(2):397‐401. 30887798

[20] Hybbinette S , Boström M , Lindberg K . Enzymatic dissociation of keratinocytes from human skin biopsies for in vitro cell propagation. Exp Dermatol. 1999;8(1):30‐38. 10206719 10.1111/j.1600-0625.1999.tb00345.x

[21] Ścieżyńska A , Nogowska A , Sikorska M , et al. Isolation and culture of human primary keratinocytes‐a methods review. Exp Dermatol. 2019;28(2):107‐112. 30548893 10.1111/exd.13860

[22] Rheinwald JG , Green H . Serial cultivation of strains of human epidermal keratinocytes: the formation of keratinizing colonies from single cells. Cell. 1975;6(3):331‐343. 1052771 10.1016/s0092-8674(75)80001-8

[23] Daniels JT , Kearney JN , Ingham E . Human keratinocyte isolation and cell culture: a survey of current practices in the UK. Burns. 1996;22(1):35‐39. 8719314 10.1016/0305-4179(95)00085-2

[24] Chen RH , Zhu J , Zhang RZ , Wang SY , Li Y . The tolerance of human epidermal cells to trypsinization in vitro. Cell Tissue Bank. 2020;21(2):257‐264. 32103403 10.1007/s10561-020-09818-3

[25] Reddy NM , Lange CS . Serum, trypsin, and cell shape but not cell‐to‐cell contact influence the X‐ray sensitivity of Chinese hamster V79 cells in monolayers and in spheroids. Radiat Res. 1991;127(1):30‐35. 2068269

[26] Xie Z , Shi J , Zong M , et al. Isolation and culture of primary human gingival epithelial cells using Y‐27632. J Vis Exp. 2021;177:e62978. 10.3791/62978 34806697

[27] Hsueh YJ , Huang SF , Lai JY , et al. Preservation of epithelial progenitor cells from collagenase‐digested oral mucosa during ex vivo cultivation. Sci Rep. 2016;6:36266. 27824126 10.1038/srep36266 PMC5099970

[28] McAtee CO , Barycki JJ , Simpson MA . Emerging roles for hyaluronidase in cancer metastasis and therapy. Adv Cancer Res. 2014;123:1‐34. 25081524 10.1016/B978-0-12-800092-2.00001-0 PMC4445717

[29] Kramer MW , Golshani R , Merseburger AS , et al. HYAL‐1 hyaluronidase: a potential prognostic indicator for progression to muscle invasion and recurrence in bladder cancer. Eur Urol. 2010;57(1):86‐93. 19345473 10.1016/j.eururo.2009.03.057 PMC2828527

[30] Caffesse RG , Nasjleti CE . Enzymatic penetration through intact sulcular epithelium. J Periodontol. 1976;47(7):391‐397. 181555 10.1902/jop.1976.47.7.391

[31] Adebowale K , Gong Z , Hou JC , et al. Enhanced substrate stress relaxation promotes filopodia‐mediated cell migration. Nat Mater. 2021;20(9):1290‐1299. 33875851 10.1038/s41563-021-00981-w PMC8390443

[32] Driehuis E , Kolders S , Spelier S , et al. Oral mucosal organoids as a potential platform for personalized cancer therapy. Cancer Discov. 2019;9(7):852‐871. 31053628 10.1158/2159-8290.CD-18-1522

[33] Calenic B , Greabu M , Caruntu C , et al. Oral keratinocyte stem/progenitor cells: specific markers, molecular signaling pathways and potential uses. Periodontol 2000. 2015;69(1):68‐82. 26252402 10.1111/prd.12097

[34] Taniguchi Y , Nagao T , Maeda H , et al. Epithelial cell proliferation in oral lichen planus. Cell Prolif. 2002;35 Suppl 1(Suppl 1):103‐109. 12139713 10.1046/j.1365-2184.35.s1.11.x PMC6496840

[35] He Z , Feng M . Activation, isolation, identification and culture of hepatic stem cells from porcine liver tissues. Cell Prolif. 2011;44(6):558‐566. 21988556 10.1111/j.1365-2184.2011.00781.x PMC6496005

[36] Tuveson D , Clevers H . Cancer modeling meets human organoid technology. Science. 2019;364(6444):952‐955. 31171691 10.1126/science.aaw6985

[37] Voskoglou‐Nomikos T , Pater JL , Seymour L . Clinical predictive value of the in vitro cell line, human xenograft, and mouse allograft preclinical cancer models. Clin Cancer Res. 2003;9(11):4227‐4239. 14519650

[38] Johnson JI , Decker S , Zaharevitz D , et al. Relationships between drug activity in NCI preclinical in vitro and in vivo models and early clinical trials. Br J Cancer. 2001;84(10):1424‐1431. 11355958 10.1054/bjoc.2001.1796 PMC2363645

[39] Jiang Q , Yu Y , Ruan H , Luo Y , Guo X . Morphological and functional characteristics of human gingival junctional epithelium. BMC Oral Health. 2014;14:30. 24708739 10.1186/1472-6831-14-30 PMC4234347

[40] Mendonsa AM , Na TY , Gumbiner BM . E‐cadherin in contact inhibition and cancer. Oncogene. 2018;37(35):4769‐4780. 29780167 10.1038/s41388-018-0304-2 PMC6119098

[41] Tan SH , Barker N . Wnt signaling in adult epithelial stem cells and cancer. Prog Mol Biol Transl Sci. 2018;153:21‐79. 29389518 10.1016/bs.pmbts.2017.11.017

[42] Menon SS , Guruvayoorappan C , Sakthivel KM , Rasmi RR . Ki‐67 protein as a tumour proliferation marker. Clin Chim Acta. 2019;491:39‐45. 30653951 10.1016/j.cca.2019.01.011

[43] Goldhammer N , Kim J , Timmermans‐Wielenga V , Petersen OW . Characterization of organoid cultured human breast cancer. Breast Cancer Res. 2019;21(1):141. 31829259 10.1186/s13058-019-1233-x PMC6907265

[44] Bragulla HH , Homberger DG . Structure and functions of keratin proteins in simple, stratified, keratinized and cornified epithelia. J Anat. 2009;214(4):516‐559. 19422428 10.1111/j.1469-7580.2009.01066.x PMC2736122

